# Limiting and facilitating access to innovations in medicine and agriculture: a brief exposition of the ethical arguments

**DOI:** 10.1186/s40504-014-0008-5

**Published:** 2014-04-05

**Authors:** Cristian Timmermann

**Affiliations:** Applied Philosophy Group, Department of Social Sciences, Wageningen University, Hollandseweg 1, 6706 KN Wageningen, The Netherlands; Centre for Society and the Life Sciences, PO-box 9010, 6500 GL Nijmegen, The Netherlands

**Keywords:** Global justice, Intellectual property, Benefiting from science, Human rights, Access to medicines

## Abstract

Taking people’s longevity as a measure of good life, humankind can proudly say that the average person is living a much longer life than ever before. The AIDS epidemic has however for the first time in decades stalled and in some cases even reverted this trend in a number of countries. Climate change is increasingly becoming a major challenge for food security and we can anticipate that hunger caused by crop damages will become much more common.

Since many of the challenges humanity faced in the past were overcome by inventive solutions coming from the life sciences, we are compelled to reconsider how we incentivize science and technology development so that those in need can benefit more broadly from scientific research. There is a huge portion of the world population that is in urgent need for medicines to combat diseases that are currently neglected by the scientific community and could immensely benefit from agricultural research that specifically targets their environmental conditions. At the same time efforts have to be made to make the fruits of current and future research more widely accessible. These changes would have to be backed by a range of moral arguments to attract people with diverging notions of global justice. This article explores the main ethical theories used to demand a greater share in the benefits from scientific progress for the poor. Since life sciences bring about a number of special concerns, a short list of conflictive issues is also offered.

One of the most fundamental norms in human rights law is the principle of progressive realization of all human rights.^a^ Perhaps the most incontrovertible and shared standard to measure progress towards achieving this goal is life expectancy. Since the 1950s a substantial rise in life expectancy can be observed all around the world. Contributions to this remarkable achievement come from a wide range of quarters; however a crucial role can be attributed to the rapid development of the life sciences. In the early post-war period a number of newly developed innovations in the life sciences were made widely available, contributing to the eradication of some dreadful diseases and making an immense increase in harvest yields possible.

Current developments are however challenging the progressive realization of human rights worldwide. At the end of the 20^th^ century, for the first time in decades, not war but disease was responsible for lowering the average life expectancy in a number of countries (World Health Organization 2012). The AIDS pandemic has had such a devastating death toll that is reflected in a considerable reduction of the national average life expectancy of various countries. In addition, hunger and malnutrition are endemic; many experts prognosticate that climate change will impose a further threat to future food security (Cline 2007). The state of some countries is so dire that the everyday term “developing country” does not fit current realities any more. For many countries the label “retrogressive societies” would be more suitable to describe the actual situation.

The reduction of average life expectancy in many countries is an undisputable sign that one of the most sacred principles of human rights law, the principle of progressive realization, is being violated, demanding urgent response. All disciplines capable of delivering solutions incur a moral obligation to contribute to the alleviation of these problems.

Science and technology development have still a huge potential to address many of the welfare issues currently at stake.^b^ Optimal results can be reached when research agendas are aligned to meet the needs of the worst off. Unfortunately, research efforts destined to meet the global poor’s needs are insufficient and many of the inventions that could help addressing the problems of the poor are priced out of their financial reach. The scientific community is currently neglecting a number of diseases and much research still needs to be done to improve tropical agriculture.

Those problems are partly due to the fact that it has become customary to incentivize research and development by granting temporary exclusive rights to innovators. Many vital medicines and innovations in agriculture are subject to those rights and sold at higher than production prices to allow innovators to recoup research and development costs. Extreme inequality makes the development of products for the desires of the rich much more profitable than it is to address the problems of the poor.

Since allowing the global poor to benefit from scientific and technological development in agriculture and medicine more widely can play such a crucial role in improving human welfare, we will first examine the main arguments that defend temporary exclusivity. As innovation in the life sciences brings about a series of extra considerations that need to be addressed, we will briefly list some of these issues in the second part. The third section will analyse the ethical theories used to bring research agendas more in line with the needs of the poor and to make innovations accessible.

## Justifications for temporary exclusivity

The function of intellectual property rights is primarily instrumental: it is a societal tool to stimulate innovation. Depending upon the type of creative work a variety of incentive mechanisms have been institutionalized. Examples thereof are copyright, patents, geographic indications and plant breeders’ rights. In addition, trademarks are protected to help manufacturers maintain a clientele by providing products that retain certain quality standards and characteristics. Trade secret laws set some limitations on how far employees of one company may share their acquired knowledge with competitors.

One of the most prominent forms of intellectual property are patents. Having its present-day origin in the second half of the fifteenth century in Venice, patents were from the beginning conceived as a public-private bargain (Biagioli 2006; May 2007). Since its early days, patents were only granted to inventions that were both new and useful. The exclusive rights were also temporary and alienable, and the state retained a right to compulsory licence. Interesting is that early patent law required patent holders to use the patent (a basic working requirement) in order to retain its validity.^c^ Only this last element has not been taken over by contemporary patent law. Holders of exclusive rights are nowadays generally not required to make their inventions or work publicly available and can up to a certain extent hinder third parties to make such efforts.^d^

Granting temporary exclusive rights to innovators allows them to recoup research and development costs, provided those costs were reasonable and the product developed can be sold and finds a large enough market. Inventors who can convincingly persuade investors to advance research expenses are given a tool to secure returns to the investment and so are able to undertake their research. Those who have made a financial gain by making use of exclusive rights can, if they so wish, reinvest their capital in further research activities.

The establishment of the Trade-related Aspects of Intellectual Property Rights Agreement^e^ in 1994 introduced a system for the global enforcement of intellectual property rights with sanction possibilities for noncompliance. However, similarly to its predecessors in national laws, rights are not absolute. The idea of compulsory licenses is affirmed in the TRIPS agreement (article 31). The drafters of the TRIPS agreement as well as the signatories of the Doha Declaration acknowledge that intellectual property rights can clash with higher societal goals, most notoriously public health needs. Here signatories agreed that in case of conflict, urgent public health interests supersede private interests (Timmermann and van den Belt 2013).

The globalization of intellectual property rights was not the only event that changed the legal landscape of exclusive rights during the last century. Since the second half of last century, exclusive rights are less seen as privileges and more perceived as genuine property entitlements.^f^ This change in terminology is not a minor one, since property entitlements are far more deeply anchored in society. Opponents of exclusive rights do nowadays not only have to fight entitlements that are conceived mainly as instrumental, but also rights that terminologically fall under the umbrella of property rights. Violations of the latter right are generally perceived as less acceptable. There are two major philosophical traditions that justify ownership of property by basing the encompassed rights on natural law and personality ties respectively.^g^

### Natural rights

Following an interpretation of Locke’s material property theory, modern legal scholars have translated the notion of having a natural right to enjoy the fruits of one’s labour directly into having a natural right to intellectual property.^h^ According to this theory there is nothing we are more entitled to call our own than our own bodies. Since we mix labour, something that is inherently part of our own due to the indispensable bond to our bodies, with the material we work with, we gain an entitlement to call the thing we mix labour with our own (Locke 1689; Widerquist 2010). This is subject to two provisos: the resources we mix labour with are unowned and there is enough and as good left for others (Locke 1689; Child 1990). Retaining ownership titles is however still subject to a third proviso: non-wastage has to be avoided.^i^ Similarly to reasoning found in the early Venetian patent act, the idea that ownership titles should only cover objects that are used is present in Lockean property theories.^j^

When dealing with tangibles, it makes sense to allow ownership, since harvesting the fruits of one’s labour is hardly possible without having control over the object. Intangible objects are different however, the object itself is not consumable, i.e. it can be enjoyed by a number of people at the same time without diminishing it (cf. Attas 2008). Exclusive rights on the object are not necessary to enjoy the fruits of one’s intellectual labour, at least when considering individual use. This changes however, if under enjoying the fruit’s of our labour we also include charging monopoly rents from the use of the invented object. Recognizing property makes charging rents possible, however here we may undermine one of the main functions of property: to incentivize mixing labour with the owned object (i.e. improving the asset). As soon as the practice of rent-seeking is accepted, it becomes clear that the idea of abuse of rights has to be specified (cf. van Donselaar 2009). The natural rights tradition has close ties to the notion of desert, making issues of proportionality between benefiting from one’s own labour and gaining from the efforts of others mandatory. Exorbitant rent-seeking may disincentivize potential labourers to work on further improving the asset.

### Personality theories

According to Hegelian personality theories we as individuals own our character traits, talents and feelings (Moore 2011). While constructing or creating new objects we are expressing ourselves and certain traits of our personality become attached to the developed objects. Having control over how one’s person is perceived requires therefore also a certain power over the objects one has brought into existence. Property ownership is one way to have such control. According to Hegel the recognition and possession of property contribute to the extension of one’s personality (Hughes 1988). Exclusion would be thus justified on the grounds that one sees the desired image of one’s personality in jeopardy. Our legal tradition recognizes two elements that have strong ties to personality theories. These are the right to attribution of authorship and the right to control the integrity of one’s work as protected by copyright law.

Nonetheless, when it becomes possible to limit the diffusion of agricultural innovation or block access to essential medicines solely on these grounds, we will have to seriously question the social value of securing this individual interest. This type of reasoning is difficult to defend for objects other than artistic and literary creations.

We can trace some elements of both traditions in today’s intellectual property laws. Keeping the right to have one’s name attached to an invention, or work of art, after selling it and being granted some control over the integrity of an artistic expression are as mentioned clearly compatible with the Hegelian tradition. A clause that gives authors control over the integrity of their work may impede a swift updating of scientific textbooks – conflicting with other societal interests (cf. Atenas and Havemann 2013). Similarly, giving inventors the power to withhold an invention from the public is more inspired by the natural rights tradition than incentive theories. The public benefits rarely from inventions being kept out of reach.

Yet, the influence of these traditions has limits. Exclusive rights are only valid for a certain period of time and are not categorized as absolute – states retain the power to override these rights – a clear departure from the natural rights tradition (Sterckx 2005a).

Awareness of the philosophical roots of property theories and acknowledging that temporary exclusivity is necessary to stimulate high-priced research and development still do not explain why intellectual property rights have taken the shape they have. We have an innovation incentive system in place that applies the same rules for all, regardless of the field of technology, the purchasing power of the exclusive rights applicant or the social acceptance of past licensing behaviour. Political economy may offer us the missing argument. Incentivizing innovation with a one-size-fits-all mechanism creates confidence for innovators and their investors by conveying the impression that rules are simple and valid for all. When decisions made at the patent office are predictable, uncertainties in investments are reduced, something that is highly valued by inventors and investors.

In the next section we will shift our attention to the overall social costs of recognizing a one-size-fits-all system of intellectual property rights.^k^ A first step to understand the full consequences of such a societal decision is to examine a number of special challenges brought up by temporary exclusivity over inventions in the life sciences. As we will see, intellectual property law cannot claim neutrality by simply avoiding differential treatment of technologies. The design of the innovation incentive system has an enormous effect on the kind of technologies that will be developed, who will be able to afford these products, who is included in the innovation processes and lastly who will draw the biggest benefits.

Patents that are tightly linked to genetic resources make temporary exclusivity more problematic as often competing innovators will have to seek licenses to secure freedom-to-operate. This partial control over follow-up innovation raises a problem of justice when research output is inaccessible for the poor, the chances for start-up companies restricted and research ends up disproportionally oriented towards the wishes of the rich. The design of innovation incentive systems has to take into consideration that we live in a world of extreme inequalities. Market incentives are determined by what people are *prepared* to pay and not according to what people are *able* to pay. A system that is supposedly in place for the benefit of all should not lead to outrageous advantages for few. Exclusive rights affect a much wider group than those using and producing technologies.

## Special challenges raised by the life sciences

The privatization of knowledge in the life sciences raises a number of additional difficulties. Living organisms are evolving at different degrees and paces, ranging from millions of years to a number of generations, and constantly adapting to changing environments. Our creative minds have continuously adjusted to those fluctuations by developing new medicines, pest control methods and seed varieties. In addition, a number of new biotechnologies are developed to make human lives more comfortable. We thus have a variety of systems evolving in different directions and a legal apparatus trying to keep up with these developments.

Global trade favours a harmonized legal protection system and widely shared notions of justice demand that such system is democratically backed. Under such constraints the innovation incentive system can hardly evolve parallel to biotechnological developments and the new conditions set out by the changing environment. Biological systems and human imagination are very dynamic and evolve to a great extent autonomously following own impulses. Legal systems have to cover the vast majority of possible cases, work towards simplicity and in a globalized world also aim for homogeneity.

A second group of problems is caused by individual profit maximization. Some of the strategies pursued by economically rational agents conflict with social targets such as combating disease and malnutrition. Depending on sales and licensing practices, exclusive rights can hinder access to objects of innovation that could secure such essential rights as the human rights to health and food (ICESCR, art. 11 and 12).

While the problem of access has gained prominence, it is only one of the many issues that need to be addressed. Having exclusive rights on specific uses of genetic resources may hamper innovation if patent holders pursue a restrictive licensing behaviour. Although the principle of patents is to make knowledge more accessible by giving patent holders greater control over the knowledge they claim as their own, there are repeated cases where this is not the outcome, as mentioned earlier. Through the use of temporary exclusive rights many inventions are made inaccessible regardless of what consumers are willing to pay. Compulsory licenses are the lawmaker’s remedy for such cases, but in practice they are hardly ever pursued. The public interest has to be substantial for such type of licensing to be considered a viable option (Hollis and Pogge 2008).

The list of conflicting issues is even longer. One of the reasons why the patent system was established is to provide an alternative to trade secrecy. Temporary exclusive rights are granted to those who disclose in their patent application all necessary information to carry out (i.e. reproduce) an invention. Society recognizes temporary exclusivity in order to have access to the knowledge withheld in a patent document once the 20-year exclusivity period expires. This type of agreement is often referred to as the patent bargain (cf. Murray 2006). Some innovations in the life sciences however cut the public out of their share. As we will see in two of the following sections, a number of inventions become useless over time or are prohibited once long-term side-effects are visible. The inventor enjoyed temporary exclusivity without contributing to society useful knowledge for public exploitation.

Every field of technology and science has its special problems when it comes to patents. We will examine eight difficulties, some of which are specific to the field of life sciences: (1) the impossibility to invent around some inventions, (2) the problem of patent thickets, (3) the welfare costs of temporary delays, (4) the function and effect of biodiversity, (5) how living materials react to the environment, (6) the self-multiplication of inventions, (7) the necessity of speedy sharing of data and samples during emergencies and lastly (8) the issue of biosafety.

### Impossibility to invent around

A general additional advantage of patents is that once an invention proves profitable a number of people will be motivated to offer similar solutions. Those who do not want to acquire a license from the patent holder will attempt to provide a technical innovation that has comparable functions but is distinguishable enough to qualify as a new invention and thus also be patentable. The advantage hereof for society is that the original monopoly high prices are in practice reduced by the proliferation of competing products. This competition incentivizes the original inventor to increase the sophistication of her original product and thus further increases competition, which again brings an advantage to society.

Patents that are closely tied to uses of biological material impede competing innovators to offer similar solutions as the requirement to obtain a license becomes inevitable due to the uniqueness of genetic material. Increasingly companies are exploiting this limitation by buying patents for the sole purpose of charging exorbitant licensing fees to biotechnological firms and institutes, often stalling innovation (cf. Heller 2008).

### Patent thickets

Some objects of innovations are covered by a large number of patents. When the patents have different owners with diverging conceptions of the market and scientific value of each patent, we often encounter so-called “patent thickets”. A textbook example hereof is the “golden rice” case. Licenses for nearly 70 patents had to be cleared out before the genetically modified rice could be marketed (van den Belt 2003; Wilson 2007).

### Temporality of delays

Particularly in the life sciences what for some counts as a temporary exclusion means to many permanent exclusion. Treatment that comes late is often inefficacious. Late access to medicines or vaccines may mean death or the suffering of a disease. As far as public health and food security is concerned, it is often suboptimal to have one group having early access to an innovation and another second group to have access only after generics become available. The eradication of pathogens demands widespread coordinated action.

### Biodiversity

In order to be able to apply for intellectual property protection the object of innovation has to be stable and uniform to meet the industrial application requirement for patentability (TRIPS, art. 27.1) or to qualify for plant breeders’ rights (International Convention for the Protection of New Varieties of Plants 1991, art. 5.1). Small farmers who engage in seed exchange practices identify and select a number of plant varieties according to specific traits producing a heterogeneous output and thus adding to agrobiodiversity (cf. Louwaars 2007; Schmietow 2012). This type of intellectual work can generally not be protected through the use of intellectual property law.^l^ As a consequence, those who want to apply for exclusive rights will innovate in such a way that they produce a stable and uniform output. Further, the selling of protected seed varieties is lucrative. Economically rational sales practices will include a number of outreach programmes, lobbying activities and negotiations with food retailers to drive farmers to use commercial seed varieties. This behaviour brings genetic erosion with it, as much of agrobiodiversity gets lost when farmers discontinue to use traditional seeds (De Schutter 2011).

Biodiversity has also its negative counterpart, not only useful plants have a heterogeneous genetic makeup, but also pathogens. The same active ingredient that combats a pathogen prevalent in the developed world does sometimes not have the same efficacy with pathogens prevalent in the developing world.^m^

### Living material reacts to its environment

As mentioned, one of the arguments that justify the existence of patents is that inventors disclose a huge amount of information in patent documents (TRIPS, art. 29.1) and that this information becomes part of the public domain once the patent expires. Patent databases are therefore seen as a huge source of knowledge. In the legal, philosophical and economic discourse it is assumed that knowledge is a good of non-rivalrous consumption, meaning that knowledge can be enjoyed by as many people and for as long as desired without diminishing it (Stiglitz 2008). Living organisms are however not stable. Climate change makes many crops useless. Organisms that are combated often develop resistance to the agent with whom it fights. The consequence of the latter is that many herbicides, antibiotics, antifungi and pesticides become ineffective over time. For whoever holds a patent for such type of objects, profit-maximization would dictate to either sell to a small number of high-paying customers or to overexploit the active agent without regard for the development of resistance. In case of overexploitation, the active agent may become useless once temporary exclusive rights elapse. The public will not have an effective generic product available and be obliged to pay for a newly developed patented product. An incentive mechanism to conserve those resources is missing (Outterson 2005; Anomaly 2010). If those resources are not conserved the public misses out its share of the patent bargain: valuable knowledge entering the public domain.

### Self-multiplication

Unlike in other fields, some innovations in biotechnology have the ability to self-reproduce. A prominent case is genetically-modified plants that have genes inserted whose use is protected by patents. Who is responsible for the reproductive behaviour of plants protected by exclusive rights? In the case of a plant variety that has a patented gene sequence a much-debated court case illustrates the complexities involved. The *Monsanto Canada Inc. v. Schmeiser* case has created a severe turmoil by deciding in favour of the agrochemical company (van den Belt 2009). Even by taking proper measures, it is difficult to avoid genetic contamination, as many organic farmers experience.

### Speedy sharing of data and samples in global emergencies

The impossibility to seal national borders hermetically demands that we have responsive mechanisms to enable the swift sharing of data and samples concerning public health and food security threats in place. The world we now live in hosts many more people than ever before. Overcrowded prisons are already a public health hazard (Møller et al. 2007). In so-called “hotspots” we encounter an extremely high population density living closely together with animals. As those areas are situated mostly in tropical regions, the humidity and heat provide ideal conditions for the emergence of new pathogens.^n^ An additional threat for the containment of diseases is the high mobility of people globally, which leads to the intercontinental propagation of diseases within hours.^o^

Intellectual property has a negative effect on the spirit of free sharing. Countries who voluntarily shared samples find themselves paying huge sums of money for medication that could not have been developed without their contribution.^p^ This is felt as an injustice that demotivates people to continue to share samples without clear agreements securing returns.

### Biosafety

Inventions rarely affect only technology producers and technology users, but usually also society at large. In the case those effects are negative, efforts have to be made to contain any undesired side-effects. Being able to exclusively exploit a technology in a given timeframe can make taking risks (or being risk adverse) lucrative. Not having basic needs secured makes people more willing to take risks. When research options that are affordable and far better than nothing are abandoned because they do not meet the safety standards of the Global North, harmonization of safety standards becomes a justice problem.

Biosafety regulation also affects the abovementioned patent bargain. Data submitted to biosafety regulation agencies is increasingly considered a private good (FAO & WHO 2010) and thus rarely accessible for independent testing by non-public institutions. In a number of cases patents are being used to forbid research on biotechnologies (Biddle 2014). Once exclusive rights elapse, a much wider range of stakeholders examines the submitted data. Many pesticides that were protected by exclusive rights thus become prohibited by the time generic versions can be freely manufactured because of more extensive biosafety control. Farmers are thus compelled to buy new products that are covered by patent rights.

Increasingly biosafety tests involving human subjects are carried out in lower- and middle-income countries. When objects that are improved and developed using this data are not accessible to the average biosafety trials participant we have good grounds for morally rejecting their involvement (cf. Wenner 2013).

In this section we observed that exclusive rights in the life sciences act as an additional hurdle in allowing people to participate in science, in making the benefits of scientific advancement accessible and for encouraging scientists to follow a direction that will help to solve the problems of the worst-off. As we saw, the poor are not receiving their share on what could be considered common heritage to mankind. Major corporations are tapping resources conserved and improved in the developing world without fairly compensating indigenous communities for their input. And lastly, exclusive rights are regularly used to block competitors through expensive legal battles instead of merely to secure funds for future research that could benefit society at large (cf. Sterckx 2005b).

## Incentivizing innovation and cosmopolitan conceptions of justice

Hardly anyone would nowadays endorse Leibniz’ statement that we live in the best of all possible worlds, at least when taking political realities as constitutive. There is ample room to make this world a better place. First, we live in a world of extreme inequalities. To take an example, in 2012 the combined gross domestic product of the 846.5 million people living in the low-income countries amounted to almost the same then the one reached by the five million inhabitants of Norway.^q^ There are enough resources in the world to eradicate severe poverty. In relation to hunger, it is long-known, that misdistribution and not an absolute food shortage is the main cause of famine (Sen 1981). Second, many of the global institutional arrangements predominantly benefit the richer countries of the world and come at a significant concrete and opportunity cost for the poor (Pogge 2008, 2010). And third – the subject of this writing – science and technology could be incentivized in a way that would far better benefit those with the most urgent needs. The life sciences, being tightly linked to food security and global health, have a gigantic opportunity to develop solutions for those in need.

Reducing suffering around the world to less disgraceful levels is a Herculean task. We currently face an annual death toll of 18 million people worldwide from poverty-related causes that is largely avoidable (Pogge 2008). It is estimated that 12.5% of the world population is undernourished (FAO, WFP and IFAD 2012). Vitamin and mineral deficiencies are causing irreparable damages to the health of hundreds of millions, hindering full brain development and causing blindness.^r^ All those facts are not new, and society as a whole has developed a certain apathy to see behind those evils merely numbers.

An increased global population has also made it mandatory to live more sustainable lifestyles. Climate change is threatening future food security (Cline 2007).^s^ Rising average temperatures are enlarging the area where tropical diseases are prevalent. As those diseases are neglected in pharmaceutical research we will be confronted with a huge global health problem. Pollution is affecting many areas in the world with severe effects on public health.

Even after acknowledging that tackling those problems could take more than a generation of well-intentioned people, we can still retain a glimpse of optimism and recall some of the remarkable achievements humankind made with the help of life sciences.^t^ Over the past 50 years yields in agriculture have been increased by over 130% mainly due to the immense amount of resources spent on agricultural research (Baulcombe et al. 2009). The world is now feeding many more people than at any time in history (De Schutter 2011). Some deadly diseases, such as smallpox are considered eradicated (Flory and Kitcher 2004), polio is close to be completely eradicated and others are not a threat to human life anymore.^u^

The current institutional order is designed in such a way, that the mere participation in it makes people responsible of harming others (cf. Pogge 2008). By paying taxes and buying new products we sustain a market economy that has substantial negative effects on the poor. Nonetheless, those who come up with technological innovations are not the only ones maintaining such regimes nor can they be fully blamed for the harms the poor are facing. Out of fairness, they should not be the only ones burdened with addressing global welfare problems. Further, global poverty is not a problem caused by a single generation.

Important is to mention that it is essential to human nature to improve one’s living situation and we also recognize this as a human right (UDHR, art. 11.1). Making a single generation pay for all the negligence of past generations also raises issues of justice. Over-burdening one generation will limit their possibility to improve their own situation.

Prominent moral theories vary greatly in respect to how we should balance the short-term interests of the better-off with the urgent needs of the poor and the medium- to long-term eradication of severe poverty. We will now explore six positions that aim at redressing the current situation as a matter of justice and one approach that condemns the actual situation for corrupting the norms of science.

### Weighing benefits

People who are below a certain welfare threshold are much easier to satisfy than those who already live in prosperity. To take a very basic example: giving one euro to the person earning a hundred euros a day will not significantly enhance her well-being. On the other side, giving the same euro to one of the many people who earns one euro a day will significantly help her. This is reason enough to prefer the latter person as a recipient for most utilitarians (see generally for this type of reasoning Singer 1993). Maximizing global welfare would require distributing resources to those who can convert them in welfare – either for themselves or for others – more efficiently (cf. Derclaye 2012). People who are in severe distress can already be helped with minor attentions. Having reached a threshold of welfare, people become increasingly less efficient in transforming resources into happiness (Derclaye 2013).

Transferring this principle to the subject of innovation, a thinker like Peter Singer would condemn the situation where research efforts are spent to produce an additional shaving cream for an already large menu of product choices, while diseases that afflict the lives of millions of people receive hardly any scientific attention. A situation where 90% of the global resources are spent in addressing the health problems of 10% of the world population becomes unacceptable, as it is a highly inefficient form of increasing aggregated global welfare (Drugs for Neglected Diseases Working Group 2001).

### Compensatory duties

Our global trade regimes, especially the TRIPS agreement, disproportionally benefit the developed world while adding significant disadvantages for the poor. The democratic legitimacy of the TRIPS agreement has been severely criticized. The negotiation documents were so complex that they could hardly be analysed by countries lacking strong legal expertise (cf. Drahos and Braithwaite 2003; Pogge 2008). With this treaty, Pogge argues, developed countries have made themselves guilty of imposing a harmful regime on others, thus violating the negative duty not to inflict harm. Continuing with politics as usual demands from us compensatory duties. Therefore we are obliged to establish institutions whose positive effects outweigh the negative effects caused by existing institutions. Thomas Pogge’s most known example of such type of institutions is the Health Impact Fund (Hollis and Pogge 2008). This proposal seeks to collect sufficient funds to remunerate pharmaceutical companies through a mechanism that maximizes the quality-adjusted life years of newly developed medicines. Through this fund accessibility and availability of medicines could be improved.

A similar line of thinking is prevalent in climate change negotiations. Harming others through carbon emissions is seen as inevitable. Nevertheless harming without compensating is judged as worse than harming while compensating. The transfer of technology is often presented and demanded as a form of compensation.

### Basic rights

Before being able to enjoy a wider set of liberties it is necessary to have some basic needs met. Subsistence, security and liberty are all elements that fall under the category of basic rights (cf. Shue 1996).^v^ Entitlements such as freedom from hunger and disease are examples of those rights. The basic rights doctrine aims at securing subsistence needs at a very elemental level, standards well below thresholds aimed by the International Bill of Rights.^w^ While freedom from hunger is targeted by the basic rights doctrine, the International Covenant on Social, Economic and Cultural Rights article 12.1 seeks to guarantee not only a freedom from hunger, but also a right to adequate food. An official comment on the right to adequate food states explicitly that this right shall “not be interpreted in a narrow or restrictive sense which equates it with a minimum package of calories, proteins and other specific nutrients” (UN Committee on Economic, Social and Cultural Rights 1999). This comment states that cultural and consumer acceptability should be taken into consideration (idem). Similarly the right to health is also not interpreted as freedom from disease, but as the right to the highest attainable standard of physical and mental health (cf. UN Committee on Economic, Social and Cultural Rights 2000).

A person can start to regularly enjoy other rights once her basic rights are considered secured. The basic rights doctrine seeks to secure the fundamental freedoms and entitlements for a person to be able to play a constructive role in society, without taking into consideration if the played role is the one the individual had in mind or wishes to continue to play. Important is here that the person is physically able to undertake this function. However, limiting duties to safeguard only such basic necessities is strongly criticized. We do not need to abide by such extreme positions. As mentioned earlier, in relation to food Amartya Sen has demonstrated that not scarcity but misdistribution is the principal cause of famine (Sen 1981). There are enough resources to considerably expand the freedoms people can pursue.

As far as innovation is concerned, the basic rights doctrine is a powerful tool to argue that access to some innovations takes precedence over the material interests of innovators. There are however some limitations. The link between the object of innovation and the intended outcome has to be strong, e.g. as far as health is concerned a medicine has to be crucial to recover from a disease. Objects that would considerably improve living conditions but are not vital for subsistence would still have to be balanced with other rights and interests. As long as an object of innovation is necessary to ensure subsistence society can make claims on it, this counts also for objects that become available in the future. The World Health Organization, working with a concept of essential medicines, still demands access to new medicines and repeatedly states the need for further research. The Organization constantly reviews its list, taking into consideration the state of knowledge and innovation as well as the propagation of pathogens and disorders (WHO Expert Committee on the Selection Use of Essential Medicines 2012). When objects of innovation are merely helpful, but not indispensable to reach an intended outcome, as is the case with most of agricultural innovation, the basic needs doctrine loses strength.

### Human rights and capabilities

The human rights discourse and the capabilities approach are interested in securing considerably more than just subsistence needs and freedom from repression. The two approaches have some differences, but offering opportunities to develop for people and communities is central in both discourses (cf. Nussbaum 1997). Development is understood in terms of human flourishing and this in multiple dimensions. People should also have the opportunities to participate in scientific and cultural life, benefit from the advancement of science, express themselves freely, enjoy leisure time, have a say on matters that affect their lives, among others. When addressing such an ample catalogue of rights, the opportunities innovation has to secure these rights will also be much larger (cf. Sunder 2012). Access to many more objects can be claimed in virtue of their ability to enhance full human functioning. A wider catalogue of rights that have to be pursued as a whole also enlists science to fulfilling many more tasks. It strongly demands solutions for the problems that afflict the needy (cf. Korthals and Timmermann 2012).

Living in a world where science and technology play such an enormous role also creates an ethical obligation to make science and the development of technologies a more inclusive endeavour (Timmermann 2013). Participating in such endeavours allows people to get appropriate knowledge to judge those projects and assess some of the risks involved. Educating a sufficient number of citizens to such expertise is vital for society’s self-determination. Science is also part of cultural life and as such human rights law protects a right to participate in such endeavours (Shaheed 2012). Using intellectual property to control follow-up innovation and to limit the possibilities to participate in the life sciences is therefore condemned.

It becomes important to recall that the International Bill of Rights has global legitimacy. The rights enshrined in the International Covenant on Political and Civil Rights, the International Covenant on Economic, Social and Cultural Rights and the Universal Declaration of Human Rights are based on agreement. A general agreement on the fundamental rights and entitlements of all human beings should help each individual to pursue their ideal of a good life in harmony with others worldwide. Extensive as those rights are, extensive is the agreement on people being entitled to them. The lists of central human capabilities do not count with such democratic legitimation, but nonetheless many of the targets identified by capability theorists have their pendants in human rights law (Nussbaum 1997; Marks 2011).

Human rights law and many international organizations have been very clear on the importance of technical and scientific international cooperation (e.g. regarding food security, see ICESCR, art. 11.2). The harms that afflict the poor and major environmental problems should not be seen as problems that solely perturb the countries where those issues are present.^x^

### Recognition theories

The fundamental concept behind recognition theories can be found in Hegel’s memorable words “they recognize each other as mutually recognizing one another” (Hegel 1807/1970). It is hardly surprising, then, that this small statement has attracted the attention of countless scholars. According to an interpretation of this passage, action that nurtures recognition has to be simultaneous, reciprocal, transitive, reflexive and symmetrical (Limmer 2005). Hence, a distinctive trait of this approach is the possibility to condemn relations of dependency and of one-sided influence. A contemporary representative of this tradition, Nancy Fraser, has specified the importance of being able to participate as a peer (Fraser 1998). In the context of science and technology development this can be interpreted as making research efforts more inclusive, in formally recognizing parties that where vital in bringing out a new product and in not systematically discriminating certain research contributions without good arguments. Therefore, recognition theories are a powerful tool to demand that inventive capacities of indigenous communities are publicly recognized (cf. Heins 2008; Dübgen 2012). Making the possibility of mutual influence imperative provides a justification for capacity-building efforts.

### (Re)claiming the commons

In the realm of science and technology, innovation rarely comes out of thin air (Hettinger 1989). Access to prior knowledge and data is vital for the inventive mind and we greatly rely on what previous researchers have observed, catalogued, described, refuted, discovered and invented. Setting boundaries to the use of knowledge and biomaterials by granting exclusive rights becomes increasingly restrictive for competing researchers. Many researchers from poorer institutes or those whose research area has a high patent density have insurmountable hurdles to overcome. Creative artists encounter similar constraints when making remixes or collages. This general problem has motivated a number of scholars to defend the public domain and common-pool resources, in order to secure the “building blocks” for future creativity.^y^ Those “building blocks” are essential for the continuous improvement of living conditions and to secure creative liberty. The defence of a commons of genetic resources has received much scholarly attention during the last years (see Kloppenburg 2010; Lemmens 2013; Deibel 2014).

### Scientific values

A widely shared conception of the so-called scientific ethos has been propounded by Robert Merton. He mentions four elements: communism (later communalism, in the sense of being community-centred), universalism, disinterestedness and organized scepticism (Merton 1973; van den Belt 2010). Especially philosophers of science have been accusing intellectual property for corrupting the scientific ethos. Intellectual property allows one to block access to the datasets on which one’s scientific contribution is based. Copyright protection enables journal publishers to charge high prices for subscriptions. Lack of access to new scientific contributions limits the possibility of universal validation (Biddle 2014). The principle of universalism demands validation outside one’s close circle of colleagues. A second demand of the principle of universalism is that careers be open to talents. Intellectual property fosters an environment where research avenues are barred, making it much more difficult for outsiders to prove themselves as talented. The ideal of communism calls for a common ownership of research results. It highlights the importance previous findings have for future knowledge production. Recognition and esteem of individual contributors is something that is still considered prudential, since they function as incentives. Here again the power patent holders have to control follow-up innovation is something that is condemned by followers of this tradition. They further point out that both the ideals of disinterestedness and organized scepticism are difficult to follow when financial stakes are high (Flory and Kitcher 2004). By making specific scientific innovations profitable, intellectual property creates an environment where people over- or undervalue certain inventions for other than scientific reasons.^z^

In the benefiting from scientific and technological progress context the Mertonian scientific ethos demands research results to be accessible for all, not forgetting research institutes in the developing world. Knowledge should not be locked-up in order to maintain the profitability of obsolete products or second-best solutions. Arguably, some followers of this scientific ethos would also demand a fairer evaluation of the value of traditional knowledge.

## Conclusion

This article examined the main arguments used to justify knowledge exclusivity, that is, to withhold knowledge from the public domain (even if “only” temporarily). Thereafter, some peculiarities that have to be taken into consideration when dealing with the life sciences were addressed. The third section discussed the central ethical theories used to argue for a wider distribution of the benefits from scientific progress.

A central problem of intellectual property law is that especially after the 1994 TRIPS agreement and subsequent bilateral trade negotiations it has become (almost) neutral towards the technological development it stimulates. However people have ethical judgements on the goods science and technology development is delivering, as well as on the circumstances under which these goods are being produced.

First, while there is a broad agreement on the idea that innovators should benefit from intellectual labour, the public nevertheless is interested in maintaining a balance between deserved profits and the taking of immoderate advantages. As discussed earlier, especially in biotechnology patent holders can block innovations from reaching the public without delivering equivalent alternatives. Yet civil society generally rejects any practices that resemble the establishments of cartels, such as limiting market competition, abusive pricing and blocking fair opportunities for newcomers. Further, objects of innovations in the life sciences not only satisfy consumers’ wishes, but often are necessities for the avoidance of hunger and disease. Exorbitant profits come seldom without casualties.

Concerning the second issue, the public is also not neutral towards what science is producing. The time where scientists could serenely satisfy their curiosity is long gone, if it ever truly existed (cf. Kitcher 2004; Reynolds 2010). Life sciences’ past successes have engendered in the public the idea that science can do much more to alleviate the huge welfare problems we currently face around the globe. Both self-interest and sympathy towards the worst-off motivate people to fight the current *status quo*. In a globalized world we share a number of hazards that do not respect national borderlines: infectious diseases, plant pathogens and climate change. Extreme poverty in one place of the world can lead to a public health hazard in the opposite part of the world. As far as sympathy towards the needy concerns, different notions of justice vary in great extent to what degree and at what speed we should address extreme deprivation. And, as mentioned earlier, some theories of justice do not content themselves in the absence of suffering but are actively set to promote well-being.

It is difficult to say which ethical theory or normative standpoint should be favoured. This is not necessary a negative outcome – the advocacy of a wide range of theories has led to a very fruitful debate. A plurality of ethical approaches is the best way to do justice to the heterogeneity in human needs and cravings as well as the complexities within the fields of science and technology (cf. Resnik 2003).

Nonetheless, we have good reasons to promote a more inclusive innovation system. Under some circumstances the strategic use of patents allows companies to block the advancement of science and competing innovators. We can safely assume that as long as it is economically rational for single companies to block innovation, it will continue to happen. This type of behaviour goes strongly against a widely shared human value: equality of opportunity.

When aiming to include a wider range of people in scientific enterprises, the capabilities and human rights approach supported by some elements of recognition theories have a strong potential (Timmermann 2013; Papaioannou). Building up research capacities is not only important as it allows people to participate in a fundamental part of cultural life (i.e. the culture of science), but also for a variety of instrumental reasons. Enabling more people to participate in science and technology development around the world increases the chances that technologies that are culturally and socially acceptable and adequate are produced. The geographic diversification of research outlets allows the manufacture of complex marketable products bringing larger revenues to the different countries. The use of market models to finance research and development in the life sciences demands that countries that import the resulting goods are fabricating products of similar commercial value to sustainably acquire these essential goods.^aa^

Whatever approach one considers as prudent, it is important to extensively engage with a central issue mentioned earlier: we live in a world of extreme inequalities. This has enormous consequences for the poor. The poor do not only suffer from being poor, but also from being so much poorer than the rich: as Thomas Pogge notes, researchers from poorer countries have already started to shift agendas to address richer markets.^bb^ Satisfying sophisticated appetites of people living in the developed world is economically much more profitable for commercial entities than addressing the urgent needs of the poor. The developed world has a huge advantage due to its technological head-start. Established research networks and sophisticated patented research tools give the developed world an enormous advantage to excel in whatever field of research is discovered in the future (Timmermann and van den Belt 2012). Under such extreme inequalities hard work and ingenuity alone will not be sufficient for the Global South to catch up. A substantial change in attitude is needed. Creativity and inventiveness coming from the Global South has to be valued for its own virtues and incorporated in a global innovation system. This way both the Global South and the developed world will mutually benefit from working together.

## Endnotes

^a^See International Covenant on Economic, Social and Cultural Rights (1966, hereinafter ICESCR), art. 2.1. See also UN Committee on Economic, Social and Cultural Rights (1990).

^b^While concentrating here on intellectual property, the problem of absorbing the full potential of biotechnology is much broader, ranging from trade issues to societal trust in corporate science, an extensive literature overview is offered by Juma (2005).

^c^May (2007), referring to Mandich (1948).

^d^Patents have nowadays also new uses. A so-called destructive use of patents occurs when patent holders use their exclusive rights to hinder innovation and the diffusion of improved competing products. The goal thereof is often to keep a high demand on older profitable products, on new uses of patents see Schneider (2010) pp. 125 ff.

^e^Trade-related Aspects of Intellectual Property Rights Agreement (1994), hereinafter TRIPS.

^f^Intellectual property as an umbrella concept dates back to the 1950s. Copyright has been understood as property for a far longer time, see Hughes (2011).

^g^Some authors also justify property as a mean to secure a “just reward”. A brief exposition and criticism of this type of argument is offered by Papaioannou (2006).

^h^For the complexity of this transition, see Hughes (1988) pp. 296–329 and Drahos (1996) pp. 41-72.

^i^In the realm of intangible property we may ponder if the “recklessly suboptimal use of resources” should be understood as wastage, see Attas (2008) p. 47 and also Sterckx (2005b).

^j^The introduction of money however makes the accumulation of wealth possible without spoilage. There are strong differences in opinion about the ultimate implications of the introduction of money for Lockean property theories, see Uberti (2013).

^k^To be more precise, the one-size-fits-all mechanism in the life sciences consists of three different incentive systems: patents, plant breeders’ rights and up to a certain extent geographical indications. In some cases additional market dominance can be secured by the strategic use of database rights, depending on national legislation.

^l^In some geographic areas this type of work might be incentivized through protected geographic indications, but this will be limited to a small number of products. On geographic indications, see Raustiala and Munzer (2007).

^m^One example is the vaccine developed to fight the human papillomavirus, see Timmermann and van den Belt (2013) fn. 46.

^n^Some densely populated areas in the developed world also qualify as “hotspots”, see Jones et al. (2008).

^o^A duty to share data related to public health emergencies is defended by Langat et al. (2011).

^p^In 2007 Indonesia stopped providing flu samples because the government feared that industry in the developed world would develop vaccines without providing any returns for the country, see The Royal Society (2012) p. 18.

^q^Numbers taken from http://data.worldbank.org/ (Accessed November 10, 2013).

^r^Over 30% of the world population suffers micronutrient deficiencies, see FAO, WFP and IFAD (2012) p. 23.

^s^On problems making agricultural innovation accessible for climate change adaptation, see Timmermann et al. (2010).

^t^Systematic observations and experimentations made by people living in indigenous communities are also considered scientific unless specified otherwise.

^u^Details about the current program of worldwide polio eradication can be found under http://www.polioeradication.org/.

^v^However Henry Shue’s approach is distinctive, since he considers a right to participation also as a basic right, see idem pp. 65-87.

^w^As commonplace in the literature, I will use the term International Bill of Rights to encompass the Universal Declaration of Human Rights (1948), the International Covenant on Civil and Political Rights (1966) and the International Covenant of Economic, Social and Cultural Rights (1966).

^x^This point has been reaffirmed by a number of declarations at the turn of the century, perhaps most prominently by the UN Millennium Development Goals.

^y^cf. e.g. for music see Boyle (2008) pp. 122-159, for synthetic biology see van den Belt (2012).

^z^for examples on biomedical research, see Reiss (2010).

^aa^The relation between the right to food and purchasing power is explored in detail in De Schutter (2009).

^bb^On the Indian pharmaceutical industry, see Pogge (2008) p. 231.
